# Many Roles of Carbohydrates: A Computational Spotlight on the Coronavirus S Protein Binding

**DOI:** 10.1021/acsabm.2c01064

**Published:** 2023-03-22

**Authors:** Suman Maity, Atanu Acharya

**Affiliations:** † Department of Chemistry, Syracuse University, Syracuse, New York 13244, United States; ‡ BioInspired Syracuse, Syracuse University, Syracuse, New York 13244, United States

**Keywords:** MD simulations, spike proteins, glycans, carbohydrates, coronavirus, ACE2

## Abstract

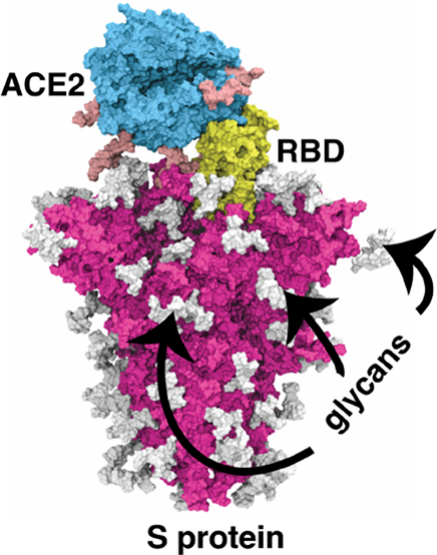

Glycosylation is one of the post-translational modifications with more than 50% of human proteins being glycosylated. The exact nature and chemical composition of glycans are inaccessible to X-ray or cryo-electron microscopy imaging techniques. Therefore, computational modeling studies and molecular dynamics must be used as a “computational microscope”. The spike (S) protein of SARS-CoV-2 is heavily glycosylated, and a few glycans play a more functional role “*beyond shielding*”. In this mini-review, we discuss computational investigations of the roles of specific S-protein and ACE2 glycans in the overall ACE2-S protein binding. We highlight different functions of specific glycans demonstrated in myriad computational models and simulations in the context of the SARS-CoV-2 virus binding to the receptor. We also discuss interactions between glycocalyx and the S protein, which may be utilized to design prophylactic polysaccharide-based therapeutics targeting the S protein. In addition, we underline the recent emergence of coronavirus variants and their impact on the S protein and its glycans.

## Introduction

**Figure 1:**
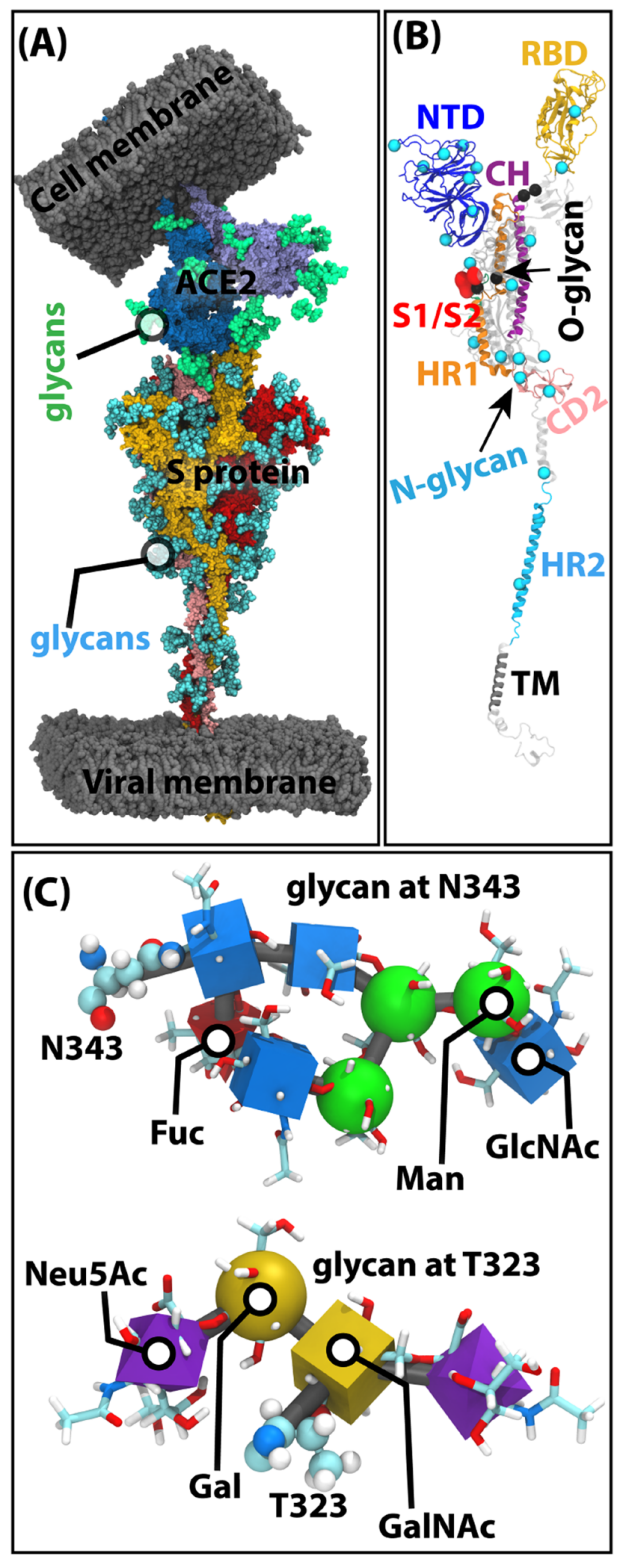
(A) Representative model of an ACE2 attached to a single RBD of the S protein. The model was constructed by combining the ACE2 dimer model from ref (14) (The model is used with permission from the authors. Copyright 2021 Amaro and co-workers) and the S-protein model from ref (10) (The data is released under a Creative Commons Attribution 4.0 International Public License. Copyright 2020 Amaro and co-workers). Each protomer of ACE2 and the S protein are shown in different colors. The ACE2 and S protein glycans are shown in green and cyan color, respectively, using VDW representation. The locations of the well-defined N-linked and a few O-linked glycans are shown in cyan and black circles, respectively. (B) Different domains of the SARS-CoV-2 S protein. The definitions of the domains are used from ref (10). (C) N-linked glycan (top) connected to N343 and an O-linked glycan (bottom) connected to T323. The structures are taken from ref (15) with permission (Copyright 2022 Gumbart and co-workers). The carbohydrates are also represented in the 3D-SNFG representations.^16^

Coronaviruses from the *Betacoronavirus* genus have been responsible for three separate epidemics in the last 20 years, i.e., SARS-CoV (SARS epidemic in 2002–2003), MERS-CoV (MERS epidemic in 2012), and SARS-CoV-2 (COVID-19 pandemic in 2019-current). Coronaviruses use the trimeric spike (S) glycoprotein, which protrudes from the surface, to latch on to a receptor on the host cell’s surface to initiate viral infection. The S proteins of SARS-CoV and SARS-CoV-2 bind to the human angiotensin-converting enzyme 2 (ACE2).^1−4^ A close relative of the MERS-CoV also binds to ACE2.^5^ The ACE2 protein is commonly found in the lungs, hearts, pancreas, intestines, and liver, providing multiple entry points for the virus.^6,7^ The S protein uses a receptor-binding domain (RBD), which transitions from a down to an up state and only binds to the ACE2 receptor in the up state (Figure 1A,B).

About 50–70% of human proteins are glycosylated.^8^ While the coronavirus S protein is highly glycosylated, it is far from being the most glycosylated protein.^9^ Glycosylation patterns are distinct in different types of viruses. Glycosylation of the S protein and the ACE2 receptor plays an important role in receptor binding and the immune escape of coronavirus. The RBD remains covered by glycans in the down state, and it becomes vulnerable to immune detection only when it opens up to bind to a receptor.^10,11^ However, the non-RBD domains of spike protein can potentially be targeted by antibodies in any conformation of the S protein.^12,13^ While current vaccines are effective against presently circulating variants of the SARS-CoV-2 virus, the threat from future variants and betacoronaviruses, in general, looms large.

Glycosylation in the S protein can occur primarily in one of two forms: N-linked or O-linked to specific protein residues (Figure 1C). The N-linked glycosylations require an Asn-X-Thr/Ser amino acid sequence, where X is any amino acid except proline. The N-linked glycan connects to the asparagine (Asn) residue through an N-acetylglucosamine (GlcNAc) sugar. Usually, the first few sugars of a glycan are GlcNAc_2_Man_3_, which are connected to subsequent sugar molecules creating an oligosaccharide chain (Figure 1C). In general, buried glycosylation sites have high oligomannose content since these sites are not easily accessible to enzymatic modifications.^17,18^ In contrast, surface glycans are readily enzymatically modified, linking other sugar molecules like GlcNAc, galactose (Gal), fucose (Fuc), and sialic acid (Sia). The O-linked glycans are connected to threonine or serine residues. Despite the possibility of many rotational conformations between two sugar molecules, the flexibility of the glycosidic bond is often restricted by the molecule’s stereochemistry and electronic effects.^17,19^ For example, although the oligosaccharide Man_9_GlcNAc_2_ has 10 glycosidic linkages, it has only four stable conformations.^17,20^ Web-based computational modeling tools like GLYCAM^21^ and CHARMM-GUI^22−24^ consider these conformational restrictions to generate realistic models of glycoproteins with site-specific glycans. Detailed reviews on the computation of conformational dynamics of oligosaccharides may be found elsewhere.^17,19,25^

The roles of individual amino acids at the interface between the S protein and the ACE2 receptor were investigated in detail.^26−30^ More and more studies are beginning to highlight the role of individual glycans. Usually, viruses utilize glycan shielding to avoid detection by the host immune systems. However, recent studies demonstrated additional functional roles of a few SARS-CoV-2 S-protein glycans^10,31,32^ and ACE2 glycans.^11,33,34^ These studies highlight the role of glycans beyond just shielding the S protein from the immune response.^10^ Cryo-electron microscopy (cryo-EM) and X-ray crystallography can not resolve glycan composition, which depends on the location of the glycan, cell type, blood groups, and environmental conditions.^35,36^ The structural studies resolve only a few sugar molecules at each glycosylation site. Mass spectrometry only provides a population distribution of glycans with different compositions at each site without revealing their functional roles.^37^ Therefore, modeling studies and molecular dynamics (MD) must be used as a “*computational microscope*” to tease out atomic details. In this mini-review, we highlight some of the computational studies on the role of glycans of the S protein and ACE2 receptor.

## Impact of S-Protein Glycans

**Figure 2:**
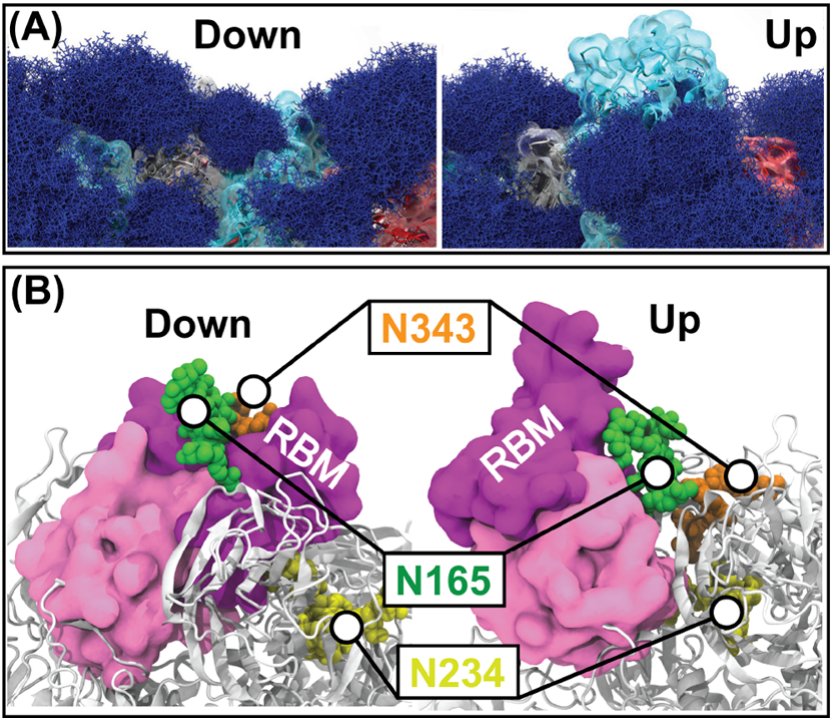
Role of S-protein glycans. (A) Glycan covers up the RBD in the down state (left), while the RBD becomes more exposed in the up state (right). The exposed area is shown in cyan. (B) S-protein glycans at N165, N234, and N343 play intricate roles in stabilizing the down and the up state. The top and bottom panels are reproduced with permission from ref (10) (Copyright 2020 American Chemical Society) and ref (15) (Copyright 2022 Gumbart and co-workers), respectively.

Overall, the SARS-CoV-2 S protein has 22 N-linked^11,38,39^ and varying O-linked glycans.^40−42^ Interestingly, SARS-CoV and SARS-CoV-2 betacoronavirus differ in the total number and positions of glycans. The S protein of SARS-CoV contains 23 N-glycosylation sites, while SARS-CoV-2 has only 22, with 18 out of 23 S-protein N-glycosylation sites preserved in SARS-CoV-2, suggesting atomic-level changes under evolutionary pressure.^9,38^ The glycans represent ∼25% of the mass^9^ and cover ∼40% of the surface area^12^ of the SARS-CoV-2 S protein. Collectively, these glycans create a protective shield on the spike protein to avoid detection by the immune system^10,11,15^ (Figure 2A). However, an average representation of the covered surface area may not be conclusive evidence of glycans’ shielding effect since some can be removed from their binding site by the antibody.^43,44^ Therefore, investigation of conformational changes of the S protein provides more detailed information about localized changes in exposure of an epitope.^15,32,45,46^ Nevertheless, even with extensive glycan shielding of the S protein, regions of vulnerabilities are highlighted by many groups.^9,10,13,15^ This section highlights more subtle roles of S protein and ACE2 glycans and cellular polysaccharides beyond shielding from the host immune response.

### S-Protein Glycans Stabilize down and up States

The glycosylation site at the N370 position is absent in SARS-CoV-2, possibly to expedite and enhance the ACE2 receptor binding.^31,33^ Computational studies highlighted the effect of this missing RBD glycan at N370 on the S protein. Using equilibrium MD simulations, Acharya et al. showed that in the absence of the N370 glycan, the ACE2 glycans could interact with the RBD more frequently.^33^ Furthermore, Fadda and co-workers demonstrated from MD simulations model S protein that the RBD opening is reduced by adding a complex glycan (FA2G2) at N370.^31^ The S-protein mutant A372T adds the glycan back at N370 and reduces the transition to the up state. The authors showed that the glycan at N370 holds the S protein in its down state by wrapping around it.^31^ Therefore, the equilibrium is shifted toward the up state in the Wuhan-hu-1 variant, which does not contain this glycan. Independent binding measurements later found that the A372T mutant of SARS-CoV-2 S protein binds more loosely to ACE2 (5-fold decrease).^47^ However, the decrease in binding affinity is not as profound in the case of A372T RBD (not the whole S protein), indicating the down-to-up dynamics is more impacted by the glycan at N370 than the actual ACE2 binding process.^47^

The glycans at N165, N234, and N343 also influence the opening and closing dynamics of RBD.^10,11,15^ In the early phase of the pandemic, a seminal study by Amaro and co-workers highlighted a few of glycan’s roles beyond just the shielding effects from antibodies.^10^ Using the reported structures of the S protein in the up^48^ and down^49^ states, the authors created model up and down states by incorporating a variety of glycans in addition to modeling the unresolved domains. Subsequent μs-scale equilibrium MD simulation of the systems revealed the behavior of the glycans at N234 and N165 in the down and up states. The glycan at N234 from the adjacent monomer occupies the void created by the open RBD in the up state, while the glycan at N165 inserts in between the open RBD and the NTD of the adjacent protomer.^10^ Similar interactions were later found in enhanced sampling studies.^15,32^ The minimum energy path (MEP) along a 2-D potential of mean force (PMF) calculated using replica-exchange umbrella sampling (REUS) reveals that the glycans at N165 and N122 disrupt the hydrogen bonds between the open RBD and the NTD of the adjacent protomer.^15^ The glycan at N343 shields the RBD surface in the down state.^32^ It also opens with RBD and lifts the RBD through multiple sequential glycan–protein interactions, creating the so-called “*glycan-gating*” effect.^32^ These interactions were deciphered from the weighted ensemble (WE)-enhanced sampling technique.^50,51^ Recently, the minimum energy path for this down–up-open transition was calculated in the presence and absence of all glycans.^15^ The authors showed that glycans at N343 and N165 stabilize the down and up states (Figure 2B). However, the RBD can open further than observed in the cryo-EM structures due to the conformational flexibility of the S protein.^32,52^ The effects of these glycans were tested experimentally by designing mutants without the glycan. The N165A, N234A, and N343A mutations remove the glycan from the respective site.^10,32^ The binding affinity of the spike protein to ACE2 decreases by 10%, 40%, and 56%, respectively.^10,32^ Thus, these three glycans are beneficial for overall spike-ACE2 binding.

Interestingly, removing all glycans from the S protein lowers the energy barrier for RBD opening, indicating an overall preference for the down state in the presence of glycans. However, more nuanced effects dictate the outcome of a binding study. For example, the overall binding process involves two sequential processes: RBD down-to-up dynamics followed by the actual binding event. The importance of these two distinct processes was recently highlighted.^15^ The authors showed that the population of the ACE2-bound S protein can plummet to only 8% if the binding free energy is increased by +2.03 kcal/mol.^15^ Therefore, the gain in the relative population of the up state in the absence of glycans can be nullified by a slight decrease in the binding affinity. Overall, removing all N-glycosylation inhibited SARS-CoV-2 infection.^53^ While the location of the glycans is essential for stabilizing the down or up states, the size of the glycans is also important.^54^ In a remarkable study, Fadda and co-workers performed equilibrium MD simulations with three different sizes (Man3, Man5, and Man9) of the glycan at N234.^31^ They found that Man3-N234 reduces the stability of the up state, while Man5 is adequate to stabilize it. However, the Man5-N234 is more dynamic in nature than Man9-N234 owing to the smaller size of Man5. Overall, the μs-scale MD simulations predict that Man3-N234 has a dominant down state population, while Man5-N234 and Man9-N234 have dominant up state populations.^31^ Several experimental studies reported the details of these glycans.^38,55,56^ Surprisingly, recent experiments showed that the nature of the glycans at N234, N165, and N343 does not change the binding affinity of the S protein to the ACE2.^57^ This apparent contradiction may originate from the distinct nature of the down-to-up equilibrium from the binding-unbinding equilibrium.^15^

Glycan–glycan and glycan–lipid interaction can also play a role in the overall dynamics of the S protein. For example, the conserved N-glycosylation site at N1193 can directly interact with lipids in the viral membrane. Furthermore, the glycans at N1173 and N1158 interact with each other. Overall, these interactions dictate the flexibility of the HR2 (Figure 1B) hinge and, consequently, the overall flexibility of the S protein head.^58^ Centrality analysis shows that the glycans at N603 and N616 are highly central in connecting the lower and upper head of the S protein.^59^ The S-protein glycans at N616 and N603 show the highest betweenness centrality (BC) values in the up and down state networks, respectively.^59^ Furthermore, the glycans at N234 and N165 also displayed high BC values in the up state, indicating their role in stabilizing the up state of the S protein.^59^

### Changes in Glycosylation Sites in Coronavirus Variants

Some glycans of the S protein have changed between SARS-CoV-2 variants. For example, the Gamma variant (P.1) has two key mutations at T20N and R190S, introducing two new glycans at N20 and N188 in this variant.^60^ Interestingly, neither mutation exists in the highly infectious Delta (B.1.617.2) variant.^60^ However, the Delta variant has a T19R mutation causing the loss of the glycan at N17, which was present in Wuhan-hu-1 and other variants. Therefore, the S protein of the Delta variants is more exposed in the NTD region. The emergence of several NTD mutations in the Delta and Omicron variants leads to significant structural changes in NTD for these variants,^61^ leading to an escape from the NTD-specific antibodies.^62^ The N188 glycan in the Gamma variant has high-mannose content. A recent computational model used a Man5 glycan at N188 and demonstrated that additional glycans increase the shielding of the S protein. Additionally, MD simulations show that the Man5 glycan at N188 occupies a deep cavity between two β-sheets formed by the down-to-up dynamics of a 13-residue loop in all three monomers.^63^ Heme metabolites usually occupy this cavity to avoid antibody immunity.^64^ In contrast, the cavity size decreases without the Man5 glycan, reducing its accessibility. Overall, the glycan at N188 in the Gamma variant is hypothesized to enhance the infectivity akin to the heme-metabolite binding process.^63^ Multiple coronaviruses show different rates of conformational change even without glycans.^65^ The SARS-CoV-2 variants show different binding energy, with the Delta variant being the strongest among Alpha, Beta, Gamma, and Delta variants when compared without glycans.^66,67^

### O-Linked Glycans of the S Protein

The N-linked glycan sites and site-specific composition analysis have been studied in detail. However, the number of O-linked glycans, site-specific compositions, and individual roles have hitherto remained unclear. In general, there is no highly specific sequence requirement for O-linked glycosylation, leading to increased difficulty in predicting the O-glycans.^42,68,69^ The total number of O-glycans varies across different experiments. For example, Shajahan et al.^39^ predicted highly populated O-glycans at T323 and S325, and Anderson et al.^70^ found O-glycans near the furin cleavage site at S673, T678, and S686. The O-glycan near the polybasic furin cleavage site may regulate the furin cleavage.^71,72^ However, earlier studies either did not find these O-glycans or estimated the populations to be very small.^11,38^ A few recent studies have reported many additional O-glycan sites. Sanda et al.,^72^ Tian et al.,^40^ and Bagdonaite et al.^73^ have reported 9, 17, and 25 O-glycan sites, respectively. Tian et al. proposed the idea of “O-Follow-N” glycosylation since 11 out of 17 of the O-glycans occur within the N+1 to N+3 site of the N-linked glycan sites.^40^ These variations in O-glycan occupation among different studies possibly arise from different conditions and sources of the S protein.^18,42,74^ For example, the O-glycosylation level differs between recombinant S1 and a trimeric S protein.^18^ An additional O-glycosylated site at T376 was observed in the Omicron variant (B.1.1.529).^75^ Data on the function of the O-glycans are even more scarce. The S-protein O-glycan at S494 increases the RBD–ACE2 binding affinity.^76^ Bagdonaite et al. found most O-glycans near unoccupied or low-populated N-glycan sites, indicating a shielding effect as the major function of the glycans.^73^ Detailed studies focusing on the role of individual O-glycans are warranted for further atomic-level insights.

### Glycocalyx Primes the S Protein to Bind to the Receptor

The glycocalyx is a collection of molecules, including glycopolymers, glycolipids, glycoproteins, and proteoglycans, attached to the surface of a cell.^77^ It plays significant roles in regulating cell morphology, membrane protein diffusion, immune system regulation, cell–cell interactions, and water permeability to name a few of its functions.^77−79^ The main difference between glycoproteins and proteoglycans is that the glycans of glycoproteins contain shorter sugar chains, usually consisting of 3–20 monosaccharides.^77^ In contrast, proteoglycans contain much longer polysaccharide chains. The most abundant component of the glycocalyx is the negatively charged polysaccharide heparan sulfate (HS), composed of repeating units of a polysulfated N-acetyl-d-glucosamine and d-glucuronic acid disaccharide.^80^ The level of sulfation and d-to-l epimerization of glucuronic acid is enzymatically controlled and may vary between samples.^80−84^ Other glycosaminoglycans (GAGs) of proteoglycans include chondroitin sulfate (CS), keratan sulfate (KS), and dermatan sulfate (DS).^85,86^ However, some are more abundant than all other GAGs depending on their location.^86^

Several viruses can utilize the glycans on the cell surface to facilitate entry into the host cell.^87,88^ Likewise, the S protein binds to the glycocalyx first for coronavirus entry into the host cell.^89,90^ This property was recently utilized to engineer the “*GlycoGrip*” assay for detecting SARS-CoV-2.^91^ Interestingly, HS seems to be required for efficient interaction between RBD and ACE2, indicating the role of HS as attachment receptors, which was proposed before the COVID-19 pandemic.^89,92−94^ The SARS-CoV-2 S protein is postulated to form a ternary complex of ACE2-spike-heparin with two separate binding sites for ACE2 and HS.^89^ The HS binding also increases the population of the up or open state, which is required for the ACE2 binding to the S protein.^89^ Thus, externally administered low-molecular-weight heparin (LMWH) molecules can compete with cellular proteoglycan binding to the S protein, leading to a prophylactic treatment option for coronavirus infections.^92,95−100^ Heparin (HEP) and HS differ by the number of substituents on the disaccharide repeating units with heparin containing more sulfates than HS because of incomplete sulfation and epimerization.^84^ HEP binds to SARS-CoV-2 S protein more strongly than SARS-CoV and MERS-CoV, with dissociation constants (*K*_D_) 73 pM, 500 nM, and 1 nM, respectively.^96^ The enhanced binding of HEP with SARS-CoV-2 has been attributed to a positively charged region on the spike protein (absent in SARS-CoV), indicating an evolutionary change in the S protein.^89^ The extent of epimerization and sulfation may control the binding strength of the HS to the S protein of the SARS-CoV-2 and its variants.^90,101,102^

**Figure 3:**
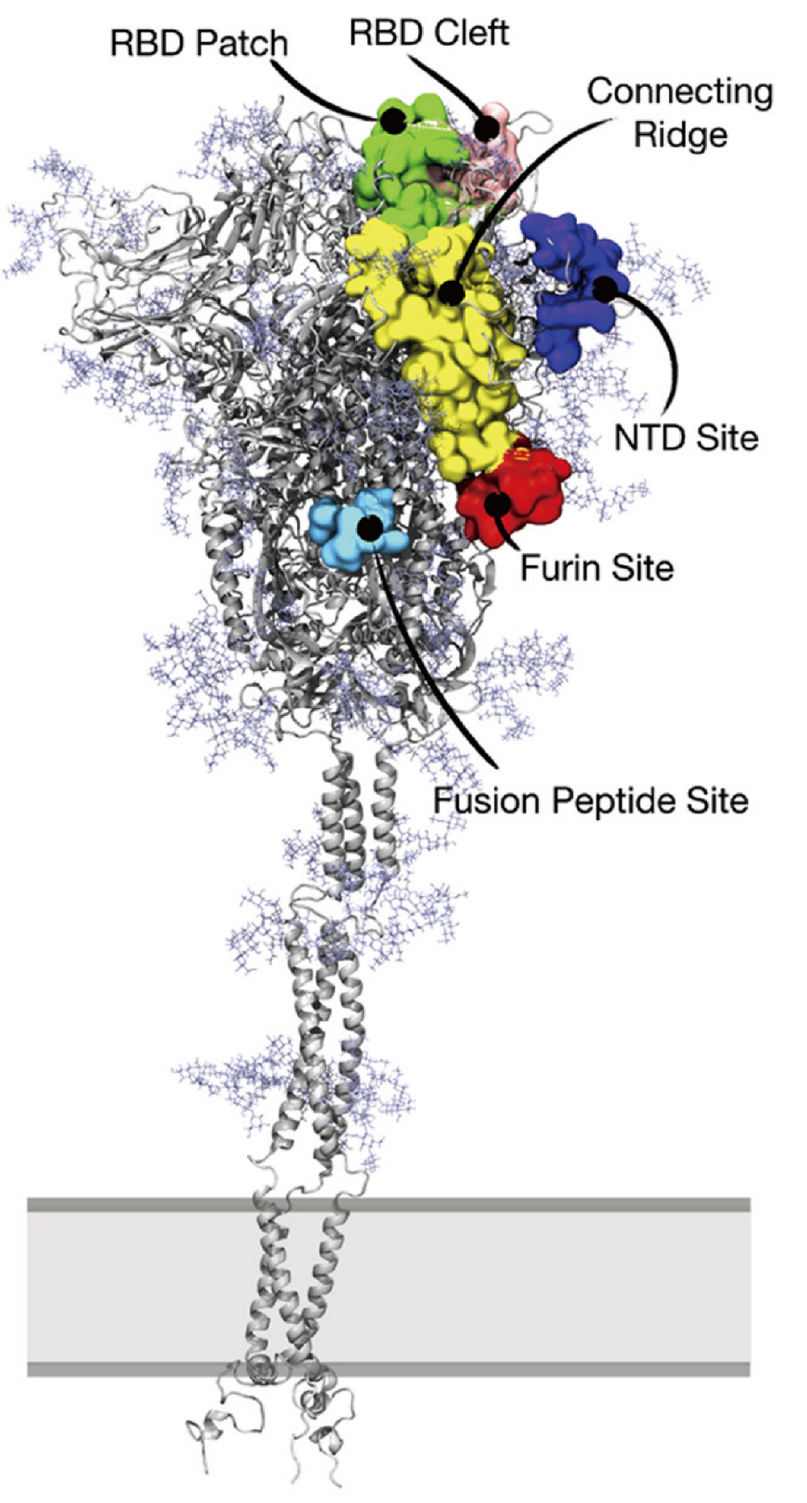
Proposed HS binding sites on the S protein. The figure is reproduced with permission from ref (91), and a few annotations have been removed for clarity. Copyright 2021 Amaro, Freeman, and co-workers. Published by the American Chemical Society.

Several binding sites of HS on the S protein have been proposed in the literature from independent docking calculations (Figure 3). Initially, HS was thought to cobind with ACE2 to the RBD of the S protein.^89,103^ Several patches with many basic amino acids form an RBD-patch binding site.^89,103^ Other important binding sites include the RBD ridge,^104^ RBD cleft,^31^ NTD,^105^ RBM,^91^ and the furin cleavage site^91^ (Figure 3). The consensus about the driving force behind S protein binding to HS is electrostatic interactions between the negatively charged HS and cluster of positive charges on the S-protein surface.^91,106−110^ In a large-scale docking study with various glycocalyx components, Kim et al. reported six novel docking sites in addition to supporting the previously proposed sites.^91^ This study investigated the 12 800 binding poses of HS-based molecules on the S protein. Owing to the long-polysaccharide structure of HS, many docking sites can be copopulated, leading to multivalent binding modes. For example, Paiardi et al. reported a trivalent docking pose where the HS occupies the polybasic S1/S2 furin cleavage site through the channel between NTD and RBD of the adjacent spike.^104^ The S-protein glycans at N122 and N165 can also occupy this ridge between the NTD and the adjacent RBD.^15^ Therefore, competition between S-protein glycans and HS for occupying a binding site is likely. Such competition is also observed for the putative binding site of the S-protein glycan at N331.^91^ In another example, the RBD cleft site can be occupied by S-protein glycans at N165 and N343^15^ or occupied by longer polysaccharide chains.^31^ In contrast, the S-protein glycans may also stabilize the HS in their binding poses while the bound HS relaxes to the conformational changes of glycans and the S protein.^91^ These effects demonstrate yet another role of S-protein glycans beyond shielding from immune detection.

With the emergence of various SARS-CoV-2 variants, the efficiency of HS binding to the S protein is also evolving. The binding affinity of HS with the S protein follows the order WT ≈ Alpha < Beta < Delta < Omicron.^102^ The total charge (without glycans) on the spike proteins (residues 13 to 1140) follows a similar trend: WT (+3) < Alpha (+6) < Beta (+15) < Delta (+18) < Omicron (+24).^102^ Since electrostatic effects control the S-protein and HS binding, the largest binding affinity is observed for the Omicron variant.^108−110^ More importantly, the charges on the S-protein head are redistributed in the Omicron variant to have a central positively charged domain, possibly facilitating HS binding.^102,111^ The total charge on the systems with a single glycosylation scheme will also have the same trend.

The kinetics of HS binding also changes between variants. Using Brownian dynamics (BD), Kim et al. showed that the HS binding to the RBD patch only occurs in the WT, and the RBD cleft is populated in WT and the Delta variant.^102^ HS never occupies both sites in the Omicron variant. However, the furin cleavage site and an RBM site are occupied by HS in WT, Delta, and Omicron variants, indicating synergy between HS and ACE2 binding.^102^ Note that the ACE2 is also highly negatively charged. The total charge on the peptidase domain (PD) of ACE2 is −26 (residues 21 to 615), calculated using models deposited by Gumbart and co-workers.^30,33^ Therefore, the overall ACE2 binding affinity to the S protein of the Omicron variant is enhanced compared to the WT S protein.^102,112^ Additionally, the Omicron evades several antibodies like STE90-C11, 4–8, S2M11, BD-368-2, and S309,^112−116^ making Omicron a highly infectious variant.

## Impact of ACE2 Glycans

**Figure 4:**
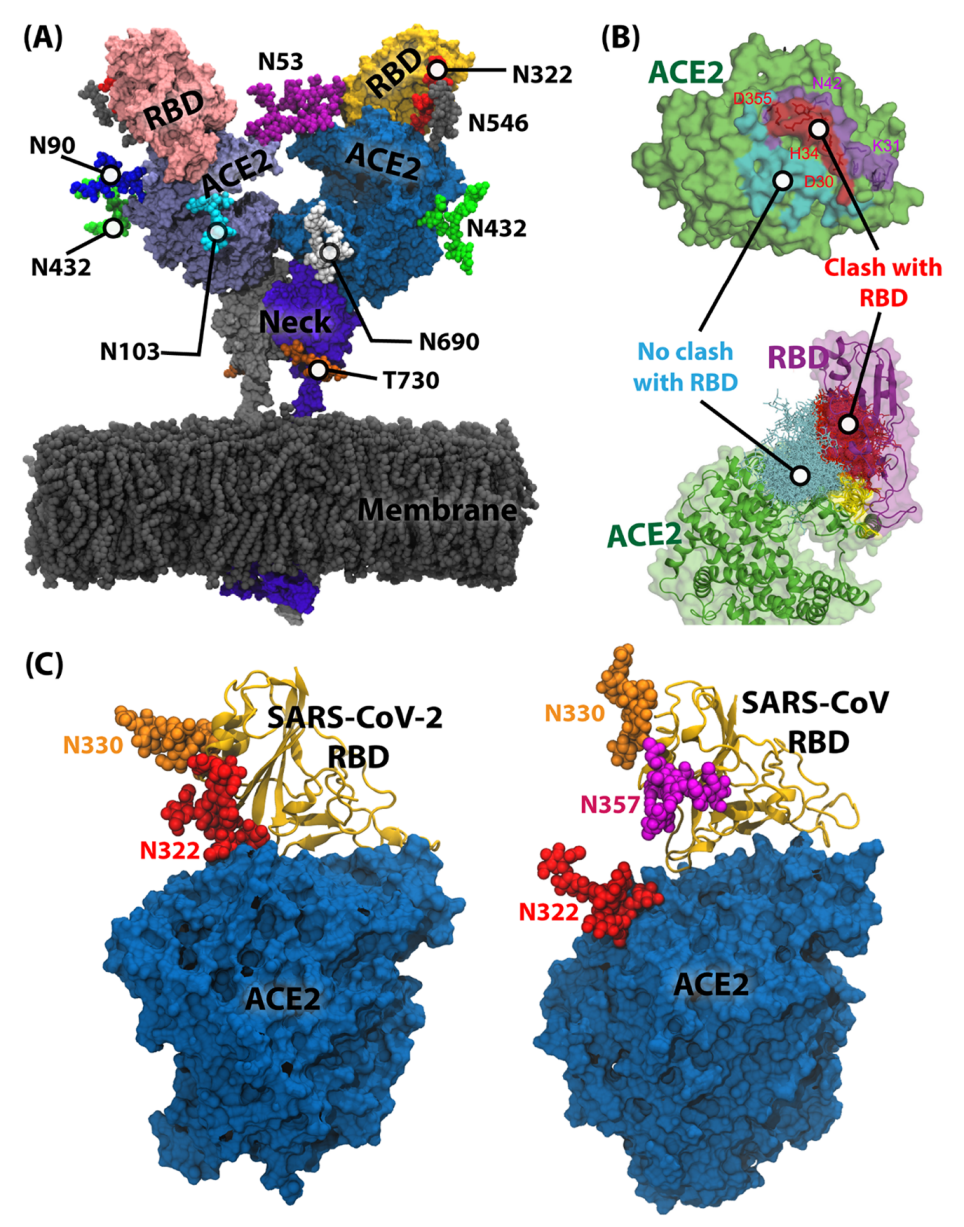
(A) Membrane-bound ACE2 dimer in complex with two RBDs represented as surfaces. The ACE2 PDs are shown in blue and ice blue, while the bound RBDs are shown in yellow and pink, respectively. The ACE2 necks are shown in violet and gray. The membrane and ACE2 glycans are shown in the VDW representation. The model used for this figure was obtained from Amaro Lab COVID-19 Data Sets associated with ref (14) (The model is used with permission from the authors. Copyright 2021 Amaro and co-workers). (B) Coverage of the ACE2 surface by the ACE2 glycan at N90 and overlap (red) of the coverage surface with the RBD binding domain (yellow). The clash-free domain is shown in cyan. This panel is adapted with permission from ref (34). (Copyright 2021 Mehdipour and Hummer). (C) Direct interaction of the ACE2 glycan at N322 with the RBD of SARS-CoV-2. The additional RBD glycan of SARS-CoV RBD at N357 (magenta) blocks this interaction. The model used for this figure was obtained with permission from the MolSSI COVID-19 database associated with ref (33) (the data is released under a Creative Commons Attribution 4.0 International Public License. Copyright 2021 Acharya, Gumbart, and co-workers).

The peptidase domain (PD) of ACE2 can bind to the RBD of S protein.^117^ Overall, a dimer of ACE2 forms a complex with the transmembrane B^0^AT1 protein. Recent MD simulations also showed that the B^0^AT1 protein does not interfere with the ACE2–RBD interactions.^34^ Furthermore, the B^0^AT1–ACE2 complex is not found in all the organs where ACE2 is expressed; B^0^AT1 is only found in kidneys and intestines.^118^ Therefore, the B^0^AT1–ACE2 complex is not necessary for ACE2 binding to the S protein. Similar to the S glycoprotein, the ACE2 receptor is also glycosylated. Since only the PD (Figure 4A) is relevant for the RBD binding, we focus on discussing the glycans of the ACE2 PD. Seven plausible N-linked and two O-linked glycosylation sites (Figure 4A) are relevant to RBD binding to ACE2. The specific glycosylation sites are located at N53, N90, N103, N322, N432, N546, N690, S155, and T730, where the S155 and T730 sites are O-glycan sites.^11,119^ However, as seen in the case of the S protein, all plausible glycan sites on ACE2 are not always occupied. For example, glycosylated S155 has a very low population.^120^ Therefore, the S155 is likely to remain unglycosylated. The N546 was also found to be 35% unoccupied.^11^ However, another study found the same site almost entirely occupied.^119^ Overall, glycosylation patterns heavily depend on the experimental conditions.^35,36^ Consequently, many different glycosylation patterns must be included in computational models to mimic realistic RBD–ACE2 systems.

### ACE2 Glycans Bind to Specific Regions of the S Protein

If an ACE2 glycan directly contacts the RBD and RBD glycans, it enhances the overall RBD–ACE2 interaction. In contrast, an ACE2 glycan may also shield the RBD-binding region of ACE2, resulting in decreased RBD binding. Additionally, some of the ACE2 glycans may interact with the other monomer in the ACE2 dimer, providing stability to the ACE2 dimer conformations. Computational studies identified the roles of a few essential ACE2 glycans from MD simulations. These simulations are usually performed without the complete S protein. The few ACE2 glycans that make direct contact with the S proteins are the glycans at N53, N90, N322, and N546.^14,33,34,121^ Steered molecular dynamics (SMD) and subsequent comparison between the RBD–ACE2 complex of SARS-CoV-2 and SARS-CoV shows that the ACE2 glycans at N90 facilitate stronger binding with the RBD of SARS-CoV-2.^121^ Removing the ACE2 glycans from the SARS-CoV-2 RBD–ACE2 complex drops the binding strength to the SARS-CoV RBD–ACE2 level.^121^

As identified by many independent models and simulations, the ACE2 glycan at N90 plays a crucial role in the RBD–ACE2 interaction.^14,33,34,121−123^ The ACE2 glycan at N90 directly interacts with the RBD residues, increasing the overall RBD–ACE2 binding interactions.^14,34^ At the same time, MD simulations of ACE2 showed that the glycan at N90 covers the area responsible for RBD binding,^34^ as shown in Figure 4B. The net result of these two opposing effects may depend on the nature of the glycan.^33^ However, in most cases, the protective effect of the ACE2 glycan at N90 prevails. Therefore, removing the glycan at N90 by introducing mutations increases RBD–ACE2 binding affinity.^124,125^ Cross-species mutational studies emphasize the protective role of the ACE2 glycan at N90. Residues in the range of 80 to 82 in rat ACE2 are NFS while MYP in humans. The introduction of a glycan at N80 would interrupt the RBD–ACE2 binding interface. Thus, introducing rat ACE2 residues into human ACE2 significantly reduces the binding of SARS-CoV and, consequently, reduces the infection.^125^

On the other hand, MD simulations show that the ACE2 glycan at N322 directly interacts with the RBD and, therefore, contributes toward the overall RBD–ACE2 binding interactions.^33,34^ It was also demonstrated using MD simulations that the ACE2 glycan at N322 interacts more preferably with the SARS-CoV-2 RBD compared to SARS-CoV. The lack of RBD glycan at N370 of SARS-CoV-2 facilitates the binding of ACE2 with SARS-CoV-2 (Figure 4C). In contrast, the RBD glycan at N357 in SARS-CoV pushes away the ACE2 glycan at N322, reducing the RBD–N322 glycan interactions.^33^ Therefore, most point mutations (at T324) that remove the ACE2 glycan at N322 decrease RBD binding.^124^ Note that since the T324 glycan is in the RBD–ACE2 interface, mutations of T324 may have detrimental effects on binding due to the lack of protein–protein and protein–glycan interactions.^126^ Furthermore, the ACE2 glycan at N322 interacts with specific residues on the RBD. Mutation of key interacting residues also reduced the RBD–ACE2 binding affinity.^127^ A few experiments show that the effect of ACE2 glycan at N322 in binding is minimal,^128^ and overall, the impact of all ACE2 glycans in the RBD binding is minimal^129,130^ because of the small entropic effect.^131^ However, site-specific glycan removal highlighted more pronounced effects of other ACE2 glycans at N53 and N90.^126,128^ These studies highlighted the importance of modeling each glycan and comparing it with a system without the specific glycan. The ACE2 glycans at N53 and N690 stabilize the ACE2 dimer by forming inter-ACE2 glycan–glycan interactions.^14^ It is also reported that some ACE2 glycans can form glycan–glycan interaction with the S-protein glycans (outside the RBD).^11^ For example, the ACE2 glycan at N546 can interact with S-protein glycans at N74 and N165, while the ACE2 glycan at N90 interacts with S-protein glycans at N165. These interactions can be observed only when the model includes the full S protein instead of just the RBD.^11^ Furthermore, multiple studies reported that the presence of sialic acid in ACE2 glycan reduces efficient interactions between ACE2 and the S protein.^126,132^ Nevertheless, SMD simulations show that the overall RBD–ACE2 interaction is more robust in the presence of glycans compared to unglycosylated systems.^121,133^

## Summary and Outlook

Modeling the S protein and ACE2 with the attached glycans has provided crucial biophysical insights into the initial virus binding process. Many groups reported simulation in the order of several microseconds to hundreds of microseconds, sufficient to capture the dynamics of the glycans. In this mini-review, we have discussed the multifaceted roles of different glycans on the S protein and the ACE2 receptor. We have also discussed the role of cellular proteoglycans as the attachment receptor and the recent push for designing proteoglycan-inspired sensors and prophylactic drugs.^89,99^

While the total (global) exposed surface area of the entire S protein may remain the same for different glycan compositions,^12^ the local exposure of an epitope will depend on the nature of the nearby glycans.^33^ New variants of concern often present modifications to the glycan behavior either through direct changes in the glycosylation sites or the nearby protein residues. One of the consequences is the changes in the exposed surface area of the S protein, leading to ineffective antibody binding to their epitope. Therefore, simulation of the newly emerging variants with their glycosylation schemes should provide valuable insights into their properties. Automated tools such as GLYCAM^21^ and CHARMM-GUI^22−24^ have made it easier to model multiple glycan schemes for glycoproteins. However, user intervention is often required to remove clashes from the model, especially in difficult cases of the S protein, where large chunks of the S protein must be modeled computationally. Therefore, careful attention must be paid to avoid unwanted *cis*-peptide and d-amino acid during the modeling steps of glycoproteins.

The glycosylation patterns in each site are often modeled with the most populated glycan from the mass spectrometry data. Since the mass spectrometry data usually provide a distribution of different glycans, making multiple models with different glycans would be insightful. Furthermore, while the N-linked glycans have attracted more attention from the computational community, the roles of the O-linked glycans are still opaque. Studies are revealing more and more O-linked glycan sites on the S protein. Therefore, we should include those in upcoming computational models and subsequent investigations into their role. The open data-sharing philosophy proposed by Amaro and Mulholland has enabled many comparisons between multiple models and glycosylation schemes.^134^

Recent studies indicated that the GAGs could bind differently to the spike protein depending on their polysaccharide-chain lengths, creating a large diversity in their structure–function property.^91^ Therefore, the binding sites of GAGs on coronaviruses require further exploration, especially since their behavior depends on the length of polysaccharide chains and a specific substituent. More substitutions on the GAG-based molecules could be modeled and docked, and a recent study indicates that GAG-based polysaccharides are more effective against the variants of concern.^102^ These remarkable developments again bring attention to computational modeling and show promise of a near-future reality of a computationally guided prophylactic drug against coronavirus.
